# Quantum Maps with Memory from Generalized Lindblad Equation

**DOI:** 10.3390/e23050544

**Published:** 2021-04-28

**Authors:** Vasily E. Tarasov

**Affiliations:** 1 Skobeltsyn Institute of Nuclear Physics, Lomonosov Moscow State University, 119991 Moscow, Russia; tarasov@theory.sinp.msu.ru; 2 Faculty “Information Technologies and Applied Mathematics”, Moscow Aviation Institute (National Research University), 125993 Moscow, Russia

**Keywords:** non-Markovian quantum dynamics, open quantum system, power-law memory, Lindblad equation, discrete map with memory, fractional dynamics, fractional derivative, fractional integral, fractional differential equation, 26A33, 34A08, 03.65.Ta, 03.65.Yz, 45.10.Hj

## Abstract

In this paper, we proposed the exactly solvable model of non-Markovian dynamics of open quantum systems. This model describes open quantum systems with memory and periodic sequence of kicks by environment. To describe these systems, the Lindblad equation for quantum observable is generalized by taking into account power-law fading memory. Dynamics of open quantum systems with power-law memory are considered. The proposed generalized Lindblad equations describe non-Markovian quantum dynamics. The quantum dynamics with power-law memory are described by using integrations and differentiation of non-integer orders, as well as fractional calculus. An example of a quantum oscillator with linear friction and power-law memory is considered. In this paper, discrete-time quantum maps with memory, which are derived from generalized Lindblad equations without any approximations, are suggested. These maps exactly correspond to the generalized Lindblad equations, which are fractional differential equations with the Caputo derivatives of non-integer orders and periodic sequence of kicks that are represented by the Dirac delta-functions. The solution of these equations for coordinates and momenta are derived. The solutions of the generalized Lindblad equations for coordinate and momentum operators are obtained for open quantum systems with memory and kicks. Using these solutions, linear and nonlinear quantum discrete-time maps are derived.

## 1. Introduction

In recent decades, the theory of open quantum systems has been actively developing (for example, see basic papers [1,2,3,4], books [5,6,7,8,9], and reviews [10,11,12]). The dynamics of open quantum systems can be described in terms of the infinitesimal change of quantum observables (or states) of these systems. This change is defined by some form of infinitesimal generator. The most general explicit form of the infinitesimal generator was suggested by Gorini, Kossakowski, Sudarshan and Lindblad in [1,2,3,4]. The equations, which describe dynamics of quantum observables and quantum states, contain derivatives of the first order with respect to time. Therefore, these equations are operator ordinary differential equations of first order in operator spaces (for example, see book [9]). Due to the use of only derivatives of the integer orders, the differential equations of integer orders cannot describe processes with memory. These processes with memory are characterized by the property of the dependence of the system behavior at a given time point on the history of its behavior at a certain past time interval. Integer-order derivatives are defined in an infinitesimally small neighborhood of a given time instant and do not take into account memory.

In mathematics, the differential equations of non-integer order and fractional derivatives of arbitrary (integer and non-integer) positive orders (for example see books [13,14,15,16,17] and handbooks [18,19]) are known. Fractional differentiation and fractional integration go back to many great mathematicians, such as Leibniz, Liouville, Riemann, Abel, Weyl, Kober, Erdelyi, Hadanard, Riesz, and have a long history from 1695 [20,21,22,23,24]. Fractional integrals and fractional derivatives of a non-integer order are, in fact, integro-differential operators forming a certain calculus, called fractional calculus. We should note that many standard properties of the first-order derivative are not realized for fractional derivatives of the non-integer order [25]. For example, a product rule—chain rule—semigroup property has strongly complicated analogs for fractional derivatives [26,27,28,29,30].

Fractional differential equations of non-integer orders, with respect to time, are powerful tools for describing processes with memory, as well as non-locality in time in various sciences, including physics [31,32], economics [33,34], and other sciences. Physical systems, which are described by fractional differential equations with derivatives of non-integer orders with respect to time, can be characterized by memory effects that correspond to intrinsic dissipative processes [35,36,37,38].

Attempts to construct a non-Markovian theory of open quantum systems with memory have been actively undertaken in recent years (see, for example, reviews [39,40,41], and articles [42,43,44], and references therein). The non-Markov character of quantum processes was often interpreted as memory effects; that is, the dependence of the dynamics at the current moment of time on the history of the system’s behavior on a finite interval in the past. All these attempts were not associated with the use of fractional calculus and mathematical theory of equations, with derivatives and integrals of non-integer orders.

For the first time, the use of fractional derivatives and integrals of a non-integer order, with respect to time, to take into account memory effects (non-Markovity) in open quantum systems was proposed in work [45] (see Chapter 20 in book [45,46,47]).

For the first time, fractional powers of Lindblad superoperators were defined and used to describe open quantum systems with memory in work [9] in 2008 (see Chapter 20 in book [9], Chapter 20 in book [45,48,49]). Solutions of generaized Lindblad equations, which describe non-Markovian quantum dynamics, were derived in works [9,45,48,49].

We also note some other possibilities for constructing a theory of non-Markovian dynamics of open quantum systems in the following directions:

1. Non-Markovian dynamics of open quantum systems with memory [46], (pp. 477–482, [45]).
2. Generalization of Markovian equations of closed and open quantum systems by using fractional power of the Lindblad superoperator [48,49,50], (pp. 433–444, [9]), (pp. 458–464, 468–477, [45]), and generalization using Grunvald–Letnikov fractional derivatives [51].
3. Non-Markovian dynamics of open quantum systems with memory and time-dependent parameters [47,52].
4. Uncertainty relation for open quantum systems [53].
5. Path integral for open quantum systems [54], (pp. 475–485, [9]).
6. Pure stationary states of open quantum systems [55,56], (pp. 453–462, [9]).
7. Open quantum system as quantum computer with mixed states [57], (pp. 487–520, [9]).
8. Relativistic open classical systems [58,59] and quantum systems with memory [60].
9. Classical system with memory as open system [61].
10. Quantization of open classical systems [62], (pp. 361–407, [9]), and [63].

Note the non-Markovian quantum dynamics are also considered in the framework of generalizations of equations for closed systems described by the Schrodinger and Heisenberg equations. For example, we can note the Schrodinger equation with fractional derivatives with respect to time [64,65,66], and the fractional Heisenberg equations [9,50], (pp. 457–466, [45]).

An important approach to description of dynamics is discrete-time maps (for example, see [67,68,69,70,71]). In classical theory, the discrete maps with memory are considered in the papers [72,73,74,75,76,77]. In these works, the form of these discrete maps with memory was simply postulated and not derived from any principles or equations. It should be emphasized that all these discrete maps with memory were not derived from any differential equations of either integer or non-integer orders. In this regard, it is important to derive discrete-time maps with memory from fractional differential equations that describe dynamical systems with memory.

For the first time, discrete maps with memory were obtained from fractional differential equations in works [78,79,80] (see also (pp. 409–453, [45]) and [81,82])). It should be emphasized that no approximations were used when obtaining maps with memory (for details, see (pp. 409–453, [45])). These discrete maps with memory are exact solutions of of the fractional differential equations with periodic kicks [45,78,79,80]. Then, this approach, which is based on the equivalence of the fractional differential equations and the discrete maps with memory, has been applied in works [34,81,82,83,84,85,86,87,88,89,90,91,92,93,94] to describe properties of the discrete maps with memory. Computer simulations of some discrete maps with memory demonstrate new types of chaotic behavior and the existence of new kinds of attractor.

The memory in discrete maps means that the present step depends on all past steps. For the first time, discrete maps with memory are obtained from the fractional differential equations of classical dynamical systems in works [78,79,80] (see also Chapter 18 in book [45]).

In the proposed paper, quantum discrete maps with memory are derived from fractional differential equations with Caputo fractional derivatives. These quantum maps with memory are obtained as solutions to the generalized Lindblad equations for quantum observables. The proposed quantum maps describe non-Markovian discrete-time dynamics of open quantum systems with memory and periodic kicks.

## 2. Generalized Lindblad Equation for Open Quantum System with Memory

The first description of processes with memory and nonlocality in time was given by Ludwig Boltzmann in 1874 and 1876 [95,96]. The first physical model with memory was proposed by Boltzmann to describe isotropic viscoelastic media. Boltzmann assumed that the stress at time t depends on the strains not only at the present time t, but also on the history of changes for τ<t. He also proposed the linear superposition principle and the memory fading principle. Boltzmann suggested the use of integro-differential equations to describe the dynamics of the isotropic viscoelastic media, whose behavior is interpreted as memory effects.

The Boltzmann uperposition principle can be expressed in the form

(1) $$\overset{t_{k}=t}{\underset{t_{k}=0}{\sum }}M\left(t,t_{k}\right)\Delta X\left(t_{k}\right)=F\left(t\right).$$

Equation (1) means that the influence of the history of process changes with memory is linearly additive. Boltzmann postulated that expression (1) is valid for all small-enough step sizes $\Delta X\left(t_{k}\right)=X\left(t_{k+1}\right)-X\left(t_{k}\right)$ (or $\Delta t_{k}=t_{k+1}-t_{k}$). If X=X(t) can considered as a continuous differentiable function of time, then Equation (1) can be represented as

(2) $$\int_{0}^{t}M\left(t,\tau \right)X^{\left(1\right)}\left(\tau \right)d\tau =F\left(t\right)$$

for continuous time case.

The dynamics of open quantum systems can be described in terms of the infinitesimal change of the quantum observable. The general explicit form of the infinitesimal superoperator (infinitesimal generator) which describes this change was proposed by Gorini, Kossakowski, Sudarshan and Lindblad in [1,2,3]. In these papers, master equations of the quantum Markovian dynamics were proposed.

The quantum Markovian equation can be writtten in the form

(3) $$\frac{dA\left(t\right)}{dt}=-\frac{1}{i\hbar }\left[H,A\left(t\right)\right]+\frac{1}{2\hbar }\overset{\infty }{\underset{k=1}{\sum }}\left(V_{k}^{*}\left[A\left(t\right),V_{k}]+[V_{k}^{*},A\left(t\right)\right]V_{k}\right),$$

where A(t) is a quantum observable, H is the Hamiltonina operator, $V_{k}$ are the Lindblad operators [9]. If $V_{k}=0$ for all k∈N, then Equation (3) gives the standard Heisenberg equation.

For the description of non-Markovian quantum processes, we can take into account a memory, which means that the behavior of the quantum observable A(t) or its derivative $A^{\left(1\right)}\left(t\right)=dA(\left(t\right)/dt$ may depend on the history of previous changes of this operators. To describe this type of behavior, we cannot use differential equations of integer orders. We need use mathematical tools that allow us to take into account the presence of memory in quantum processes.

To take into account a memory, we can consider integro-differential equations instead of differential equations of the integer orders. If we take into account the memory, then we can generalize the Lindblad equation by using the integro-differential equation

(4) $$\int_{0}^{t}M\left(t,\tau \right)A^{\left(1\right)}\left(\tau \right)d\tau =-L_{V}A\left(t\right),$$

where $L_{V}$ is defined by

(5) $$L_{V}A\left(t\right)=\frac{1}{i\hbar }\left[H,A\left(t\right)\right]-\frac{1}{2\hbar }\overset{\infty }{\underset{k=1}{\sum }}\left(V_{k}^{*}\left[A\left(t\right),V_{k}\right]+\left[V_{k}^{*},A\left(t\right)\right]V_{k}\right),$$

and M(t,τ) is a memory function. Some general properties of the memory functions are described, for example, in (pp. 3–52, [34]). For M(t,τ)=δ(t−τ), Equation (4) gives the standard Lindblad Equation (3) that described quantum Markovian dynamics without memory.

We can consider the power-law form of memory fading and power-law memory functions due to the following reason. The power-law memory function can be considered as an approximation of the generalized memory functions. In works [34,97], using the fractional Taylor series in the Trujillo–Rivero–Bonilla form [98] for the memory function, we proved that the memory M(t,τ)=M(t−τ) for a wide class of functions can be represented through the power-law kernels.

The Trujillo–Rivero–Bonilla form of generalized Taylor’s formula [98] gives the equation for the memory function

(6) $$M\left(t\right)=\overset{m}{\underset{j=0}{\sum }}\frac{c_{j}}{\Gamma \left(\left(j+1\right)\beta \right)}\;t^{\left(j+1\right)\beta -1}+R_{2,m}\left(t\right)\approx \frac{c_{0}}{\Gamma \left(\beta \right)}\;t^{\beta -1},$$

where β∈[0;1], Γ(z) is the gamma function, and

(7) $$c_{j}=\Gamma \left(\beta \right)\;[t^{1-\beta }\;\left(D_{RL;0+}^{\beta }{)}^{j}M\left(t\right)\right]\left(0+\right),$$

(8) $$R_{2,m}\left(t\right)=\frac{(\left(D_{RL,0+}^{\beta }{)}^{m+1}M\right)\left(\xi \right)}{\Gamma \left(\left(m+1\right)\beta +1\right)}\;t^{\left(m+1\right)\beta },\;\xi \in \left[0;t\right].$$

In addition to using the fractional Taylor series in time variable, we can use this series for Fourier transform of the memory funstion with respect to the frequency [99]. In order to consider a more general case of power-law fading, we will not be limited in advance by the conditions for obtaining a power-law memory function from the expansion of a more general kernel in a fractional Taylor series and condition β∈(0,1]. This is due to the fact that the power-law form of time nonlocality can be obtained by other methods [34,45,99].

Using the first term of expression (6) the memory function can be described by the following power-law form

(9) $$M\left(t,\tau \right)=\frac{1}{\Gamma \left(1-\alpha \right)}{\left(t-\tau \right)}^{-\alpha },$$

where Γ(z) is the gamma function, α=1−β>0 is the memory fading parameters for quantum system with memory. The value β=0 (α=1) corresponds to the memoryless case.

If we take into account the power-law fading memory with memory function (9), then Equation (4) is a generalization of Lindblad equation for quantum observable in the form

(10) $$\left(D_{C,0+}^{\alpha }A\right)\left(t\right)=-L_{V}A\left(t\right),$$

where $L_{V}$ is defined by Equation (5), and the operator $D_{C,0+}^{\alpha }$ is the Caputo fractional derivative with respect to time t (dimensionless variable) that is defined by the equation

(11) $$\left(D_{C;a+}^{\alpha }A\right)\left(t\right)=\left(I_{RL;a+}^{N-\alpha }A^{\left(N\right)}\right)\left(t\right)=\frac{1}{\Gamma \left(N-\alpha \right)}\int_{a}^{t}{\left(t-\tau \right)}^{N-\alpha -1}A^{\left(N\right)}\left(\tau \right)d\tau ,$$

where t∈[a,b] and $A^{\left(N\right)}\left(\tau \right)$ is the derivative of the integer order N, with respect to τ. It is assumed that $A\left(\tau \right)\in AC^{N}\left[a,b\right]$, i.e., the function A(τ) has integer-order derivatives up to (N−1)-th order, which are continuous functions on the interval [a,b], and the derivative $A^{\left(N\right)}\left(\tau \right)$ is Lebesgue summable on the interval [a,b].

For α=1, Equation (10) with (5) has the form of the standard Lindblad Equation (3).

For α is non-integer, Equation (10) defines the Markovian quantum dynamics with power-law memory.

If $V_{k}=0$ for all k∈N, then Equation (10) gives [45,46,47] the Heisenberg equation with memory

(12) $$\left(D_{C,0+}^{\alpha }A\right)\left(t\right)=-\frac{1}{i\hbar }\left[H,A\left(t\right)\right].$$

Let us consider the Cauchy problem for fractional differential Equation (10), and the initial condition is given at the time t=0 by

(13) $$\underset{t\to 0+}{\lim}A\left(t\right)=A\left(0\right).$$

The solution of this Cauchy problem can be given [45,46,47] in the form

(14) $$A\left(t\right)=\Phi_{t}\left(\alpha \right)A\left(0\right),\;\left(t\geq 0\right).$$

The quantum dynamical map $\Phi_{t}\left(\alpha \right)$ is represented by the equation

(15) $$\Phi_{t}\left(\alpha \right)=E_{\alpha }\left[-t^{\alpha }L_{V}\right],$$

where $E_{\alpha }\left[z\right]$ is the Mittag-Leffler function [100] with the superoperator argument

(16) $$E_{\alpha }\left[-t^{\alpha }L_{V}\right]=\overset{\infty }{\underset{k=0}{\sum }}\frac{{\left(-t^{\alpha }\right)}^{k}}{\Gamma \left(\alpha k+1\right)}L_{V}^{k}.$$

The Mittag-Leffler function satisfies [100] the equation

(17) $$\left(D_{C,a+}^{\alpha }\;E_{\alpha }[\lambda \left(\tau -a{)}^{\alpha }\right]\right)\left(t\right)=\lambda \;E_{\alpha }[\lambda \left(t-a{)}^{\alpha }\right]$$

for λ∈C, t>a, a∈R, and α>0 (for example, see Lemma 2.23 in book [16]).

For α=1, the superoperators $\Phi_{t}=\Phi_{t}\left(1\right)$, t≥0 are completely positive superoperators on operator algebra M. If $\Phi_{t}\left(I\right)=I$, then $\Phi_{t}$ satisfies the inequality

(18) $$\Phi_{t}\left(A^{*}A\right)\geq \Phi_{t}\left(A^{*}\right){\;\Phi }_{t}\left(A\right)$$

for t≥0, and A∈M where $A^{*},A^{*}A\in M$ (for details, see Sections 15.7 and 15.8 in (pp. 319–323, [9]).

If $\Phi_{t}$ is a superoperator such that $\Phi_{t}\left(A^{*}\right)={\left(\Phi_{t}\left(A\right)\right)}^{*}$ for all A∈M. Then $\Phi_{t}$ is positive superoperator

(19) $$\Phi_{t}\left(A^{2}\right)\geq {\left(\Phi_{t}\left(A\right)\right)}^{2}$$

that can be proed by the transformations

(20) $$\Phi_{t}\left(A^{2}\right)=\Phi_{t}\left(A^{*}A\right)\geq \Phi_{t}\left(A^{*}\right)\Phi_{t}\left(A\right)=\;={\left(\Phi_{t}A\right)}^{*}\left(\Phi_{t}A\right)={\left(\Phi_{t}A\right)}^{2}\geq 0.$$

As a result, we can state that for open quantum systems, we have the inequalities

(21) $$\left(AB\right)\left(t\right)\neq A\left(t\right)\;B\left(t\right),\;\;A^{2}\left(t\right)\neq \left(A^{2}\right)\left(t\right).$$

The quantum dynamical map, which is represented by the superoperators $\Phi_{t}\left(\alpha \right)$ with t_0, describes dynamics of open quantum systems with power-law memory. The superoperator $L_{V}$ can be considered as a generator of the one-parameter groupoid $\left\{\Phi_{t}\left(\alpha \right)|\;t\_0\right\}$ on operator algebra M of quantum observables

(22) $$\left(D_{C,0+}^{\alpha }\Phi_{\tau }\left(\alpha \right)\right)\left(t\right)=-L_{V}\Phi_{t}\left(\alpha \right).$$

The set $\left\{\Phi_{t}\left(\alpha \right)|\;t\_0\geq 0\right\}$ forms a quantum dynamical groupoid [45,46,47] which is characterized by the following properties

(23) $$\Phi_{t}\left(\alpha \right)I=I,$$

(24) $${\left(\Phi_{t}\left(\alpha \right)A\right)}^{*}=\Phi_{t}\left(\alpha \right)A,\;\left(A^{*}=A\right),$$

(25) $$\underset{t\to 0+}{\lim}\Phi_{t}\left(\alpha \right)=L_{I},$$

where $L_{I}$ is an identity superoperator ($L_{I}A=A$).

As a result, the superoperators $\Phi_{t}\left(\alpha \right)$,  t_0≥0 are real and unit preserving dynamical maps on operator algebra M of quantum observables [45,46,47].

We should note that the dynamical maps (15), which are described by $\Phi_{t}\left(\alpha \right)=E_{\alpha }\left[-t^{\alpha }L_{V}\right]$, do not have the semigroup property for non-integer values of the memory fading parameter α>0. In general, we have the inequality

(26) $$\Phi_{t}\left(\alpha \right){\;\Phi }_{s}\left(\alpha \right)\neq \Phi_{t+s}\left(\alpha \right)$$

for t,s>0 and α≠1. This property is based on the fact that the Mittag-Leffler function violated the semigroup property [101,102,103] for non-integer values of α:, and we have the inequality

(27) $$E_{\alpha }[-t^{\alpha }L_{V}]E_{\alpha }[-s^{\alpha }L_{V}]\neq E_{\alpha }[-\left(t+s{)}^{\alpha }L_{V}\right].$$

As a result, the quantum dynamical groupoid $\left\{\Phi_{t}\left(\alpha \right)|\;t\_0\right\}$ with α∉N cannot form a quantum dynamical semigroup for non-integer values of the memory fading parameter α>0. This property can be interpreted as a memory.

## 3. Linear Quantum Oscillator with Memory

Let us consider an oscillator with power-law memory. Here we use the basic assumption that the general form of a bounded completely dissipative superoperator holds for an unbounded superoperator $L_{V}$. This means that the generalized Lindblad equations for unbounded operators Q and P has the form

(28) $$\left(D_{C,0+}^{\alpha }Q\right)\left(t\right)=-L_{V}Q\left(t\right),$$

(29) $$\left(D_{C,0+}^{\alpha }P\right)\left(t\right)=-L_{V}P\left(t\right),$$

where $L_{V}$ is defined by Equation (5). We also assume that the operators H, and $V_{k}$ are the functions of the coordinate and momentum operators (Q and P) to obtain exactly solvable fractional differential equations of the order α>0. The functions $V_{k}=V_{k}\left(Q,P\right)$ and H=H(Q,P) are defined in the form

(30) $$H=\frac{1}{2m}P^{2}+\frac{m\omega^{2}}{2}Q^{2}+\frac{\mu }{2}\left(PQ+QP\right),$$

(31) $$V_{k}=a_{k}P+b_{k}Q,$$

where $a_{k}$, and $b_{k}$, k=1,2, are complex numbers. In Hamiltonian, the momentum operator means that this system is a linear oscillator with friction force proportional to the velocity.

Using the definition of $L_{V}$ in Equation (5) and the canonical commutation relations for operators Q and P, we obtain the generalized Lindblad Equation (28) and (29) for operators Q(t) and P(t) in the form

(32) $$\left(D_{C,0+}^{\alpha }Q\right)\left(t\right)=\frac{1}{m}P\left(t\right)+\left(\mu -\lambda \right)Q\left(t\right),$$

(33) $$\left(D_{C,0+}^{\alpha }P\right)\left(t\right)=-m\omega^{2}Q\left(t\right)-\left(\mu +\lambda \right)P\left(t\right),$$

where

(34) $$\lambda =Im\left(a_{1}b_{1}^{*}+a_{2}b_{2}^{*}\right),$$

where $D_{C,0+}^{\alpha }$ is the Caputo fractional derivative with respect to time t, and t is a dimensionless variable.

Equations (32) and (33) describe the exactly solvable model of non-Markovian dynamics of open quantum systems, which was first proposed in works [45,46,47], where the solutions of these equations are derived. For α=1, this model gives the standard Markovian quantum model, which was proposed in [4,12] (see also [10,11]).

To derive solutions of Equations (32) and (33), we define the matrices

(35) $$A=\left(\begin{array}{c} Q\\ P \end{array}\right),\;M=\left(\begin{array}{ll} \mu -\lambda & m^{-1}\\ -m\omega^{2} & -\mu -\lambda \end{array}\right).$$

Using matrices (35), Equations (32) and (33) take the matrix representation of the generalized Lindblad equation for quantum observables in the form have

(36) $$\left(D_{C,0+}^{\alpha }A\right)\left(t\right)=M\;A\left(t\right),$$

where $-L_{V}A\left(t\right)=M\;A\left(t\right)$.

Let us consider the Cauchy problem for Equation (36) and initial condition (13). The solution of the Cauchy problem can be represented [45,46,47] in the form

(37) $$A\left(t\right)=\Phi_{t}\left(\alpha \right)A\left(0\right).$$

The quantum dynamical map $\Phi_{t}\left(\alpha \right)$ is represented through the Mittag-Leffler function with the matrix argument

(38) $$\Phi_{t}\left(\alpha \right)=E_{\alpha }\left[t^{\alpha }M\right]=\overset{\infty }{\underset{n=0}{\sum }}\frac{t^{n\alpha }}{\Gamma \left(\alpha n+1\right)}M^{n}.$$

For α=1, this dynamical map takes the standard form

(39) $$\Phi_{t}\left(1\right)=\Phi_{t}=e^{tM}=\overset{\infty }{\underset{n=0}{\sum }}\frac{t^{n}}{n!}M^{n}$$

that describes the quantum dynamics of open system without memory.

To obtain exact expression of the solution for coordinate and momentum operators, we represent the matrix M in the form

(40) $$M=N\;F\;N^{-1},$$

where

(41) $$F=\left(\begin{array}{ll} -\left(\lambda +\nu \right) & 0\\ 0 & -\left(\lambda -\nu \right) \end{array}\right),$$

(42) $$N=\left(\begin{array}{ll} -\frac{\mu -\nu }{A_{-}} & -\frac{\mu +\nu }{A_{+}}\\ \frac{m\omega^{2}}{A_{-}} & \frac{m\omega^{2}}{A_{+}} \end{array}\right),$$

and

(43) $$A_{\pm }=\sqrt{{\left|\mu \pm \nu \right|}^{2}+{\left(m\omega^{2}\right)}^{2}},\;\nu =\sqrt{\mu^{2}-\omega^{2}}.$$

Using (40), the quantum dynamical map $\Phi_{t}\left(\alpha \right)$ is represented in the form

(44) $$\Phi_{t}\left(\alpha \right)=\overset{\infty }{\underset{n=0}{\sum }}\frac{t^{n\alpha }}{\Gamma \left(\alpha n+1\right)}M^{n}=N\left(\overset{\infty }{\underset{n=0}{\sum }}\frac{t^{n\alpha }}{\Gamma \left(\alpha n+1\right)}F^{n}\right)\;N^{-1}.\;$$

As a result, we have

(45) $$\Phi_{t}\left(\alpha \right)=N\;E_{\alpha }\left[t^{\alpha }F\right]\;N^{-1}.$$

For α=1, the map $\Phi_{t}\left(\alpha \right)$ is given in the standard form $\Phi_{t}\left(1\right)=N\;e^{tF}\;N^{-1}$.

Substituting expression (42) and (41) into Equation (45), we obtain the dynamical map

(46) $$\Phi_{t}\left(\alpha \right)=\left(\begin{array}{ll} C_{\alpha }\left[\lambda ,\nu ,t\right]+\left(\mu /\nu \right)\;S_{\alpha }\left[\lambda ,\nu ,t\right] & \left(1/m\nu \right)\;S_{\alpha }\left[\lambda ,\nu ,t\right]\\ -\left(m\omega^{2}/\nu \right)\;S_{\alpha }\left[\lambda ,\nu ,t\right] & C_{\alpha }\left[\lambda ,\nu ,t\right]-\left(\mu /\nu \right)\;S_{\alpha }\left[\lambda ,\nu ,t\right] \end{array}\right),$$

where we use the functions

(47) $$S_{\alpha }\left[\lambda ,\nu ,t\right]=\frac{1}{2}\left(E_{\alpha }\left[\left(-\lambda +\nu \right)t^{\alpha }]-E_{\alpha }[\left(-\lambda -\nu \right)t^{\alpha }\right]\right),$$

(48) $$C_{\alpha }\left[\lambda ,\nu ,t\right]=\frac{1}{2}\left(E_{\alpha }\left[\left(-\lambda +\nu \right)t^{\alpha }]+E_{\alpha }[\left(-\lambda -\nu \right)t^{\alpha }\right]\right).$$

As a result, we proved the following Proposition that describes the solution of the generalized Lindblad equation that describes non-Markovian dynamics of a quantum system with memory [45,46,47].

**Proposition** **1.** *The solutions of generalized Lindblad equations for coordinate (32) and momentum (33) can be represented as the quantum discrete-time map with memory in the form*

(49) $$Q\left(t\right)=\left(C_{\alpha }\left[\lambda ,\nu ,t\right]+\frac{\mu }{\nu }\;S_{\alpha }\left[\lambda ,\nu ,t\right]\right)Q_{0}+\frac{1}{m\nu }\;S_{\alpha }\left[\lambda ,\nu ,t\right]P_{0},$$

(50) $$P\left(t\right)=-\frac{m\omega^{2}}{\nu }\;S_{\alpha }\left[\lambda ,\nu ,t\right]Q_{0}+\left(C_{\alpha }\left[\lambda ,\nu ,t\right]-\frac{\mu }{\nu }\;S_{\alpha }\left[\lambda ,\nu ,t\right]\right)P_{0},$$

*where the functions* $S_{\alpha }\left[\lambda ,\nu ,t\right]$ *and* $C_{\alpha }\left[\lambda ,\nu ,t\right]$ *are defined by expressions (47) and (48), and*ν*is the complex parameter such that* $\nu^{2}=\mu^{2}-\omega^{2}$*.*

For λ=0, expressions (49) and (50) describe solutions of the Heisenberg equation with memory (12) that is the equation for linear oscillator with Hamiltonian (30) and a power-law memory.

For α=1, we have $E_{1}\left[z\right]=\exp\left(z\right)$, and expressions (49) and (50) describe the standard solutions [12] of the Lindblad equation without memory (α=1) in the form

(51) $$Q\left(t\right)=e^{-\lambda t}\left(\cos h\left(\nu t\right)+\frac{\mu }{\nu }\sin h\left(\nu t\right)\right)Q_{0}+\frac{1}{m\nu }e^{-\lambda t}\sin h\left(\nu t\right)P_{0},$$

(52) $$P\left(t\right)=-\frac{m\omega^{2}}{\nu }e^{-\lambda t}\sin h\left(\nu t\right)Q_{0}+e^{-\lambda t}\left(\cos h\left(\nu t\right)-\frac{\mu }{\nu }\sin h\left(\nu t\right)\right)P_{0},$$

where we use the expressions

(53) $$S_{1}\left[\lambda ,\nu ,t\right]=e^{-\lambda t}\sin h\left(\nu t\right),\;C_{1}\left[\lambda ,\nu ,t\right]=e^{-\lambda t}\cos h\left(\nu t\right),$$

where sinh and cosh are hyperbolic sine and cosine.

For non-integer values of the memory fading parameter α, we can use the Mainardi representation of the Mittag-Leffler function in the form

(54) $$E_{\alpha }\left(-zt^{\alpha }\right)=f_{\alpha }\left(z^{1/\alpha }t\right)+g_{\alpha }\left(z^{1/\alpha }t\right),$$

where

(55) $$f_{\alpha }\left(t\right)=\frac{1}{\pi }\int_{0}^{\infty }e^{-rt}\frac{r^{\alpha -1}\sin\left(\pi \alpha \right)}{r^{2\alpha }+2r^{\alpha }\cos\left(\pi \alpha \right)+1}dr,$$

(56) $$g_{\alpha }\left(t\right)=\frac{2}{\alpha }e^{t\cos\left(\pi /\alpha \right)}\cos\left[t\sin\left(\pi /\alpha \right)\right].$$

The function $f_{\alpha }\left(t\right)$ describes an algebraic decay as t→∞. The function $g_{\alpha }\left(t\right)$ describes oscillations with the exponentially decaying amplitude with rate λ(α)=|cos(π/α)| and the circular frequency Ω(α)=sin(π/α). Therefore, $S_{\alpha }\left[\lambda ,\nu ,t\right]$ and $C_{\alpha }\left[\lambda ,\nu ,t\right]$ also demonstrate this algebraic decay and oscillations with the exponentially decaying amplitude.

As a result, we can state that the quantum system (linear oscillator) with memory demonstrates power-law decay. We also should emphasize that power-law decay exists for open quantum systems with memory (λ≠0) and closed quantum systems with memory (λ=0).

## 4. Generalized Lindblad Equation with Memory and Kicks

Let us consider the generalized Lindblad equation with power-law memory and periodic kicks in the form

(57) $$\left(D_{C,0+}^{\alpha }A\right)\left(t\right)=-\frac{1}{i\hbar }\left[H,A\left(t\right)\right]+\lambda_{D}D_{V}\left[A\left(t\right)\right]\overset{\infty }{\underset{k=1}{\sum }}\delta \left(\frac{t}{T}-k\right),$$

where T is the period of perturbation by a periodic sequence of kicks, which are described by delta-functions, $\lambda_{D}$ is an amplitude of the kicks, A(t) is quantum observable, $D_{C,0+}^{\alpha }$ is the Caputo fractional derivative of the order α>0 with respect to time t (dimensionless variable). The superoperator $D_{V}$ is called a dissipator [7] and defined by the expression

(58) $$D_{V}\left[A\left(t\right)\right]=\frac{1}{2\hbar }\overset{\infty }{\underset{k=1}{\sum }}\left(V_{k}^{*}\left[A\left(t\right),V_{k}]+[V_{k}^{*},A\left(t\right)\right]V_{k}\right).$$

Fractional differential Equation (57) contains the Dirac delta-functions, which are the generalized functions [104,105]. The generalized functions are functionals on a space of test functions. These functionals are continuous in a topology on the space of test functions. Therefore, Equation (57) should be considered in a generalized sense, i.e., on the space of test functions, which are continuous. In Equation (57), the product of the delta-functions and the functions A(t) is meaningful, if the dissipator $D_{V}\left[A\left(t\right)\right]$ as a function of time is continuous at the points t=kT. We can use A(t−ε) with 0<ε<T (ε→0+) instead of A(t) to make a sense of the right side of Equation (57) for the case 0<α<1, when A(kT−0)≠A(kT+0), [90,91,92].

Let us consider a free particle with memory and interaction with the environment. The perturbation by the environment is described by a periodic sequence of kicks with the period T and amplitude $\lambda_{D}$. We assume that the operators $V_{k}=V_{k}\left(Q,P\right)$ and the Hamiltonian H=H(P) have the form

(59) $$H=\frac{1}{2m}P^{2},\;V_{k}=a_{k}P+b_{k}Q,$$

where $a_{k}$ and $b_{k}$ (k=1,2) are complex numbers.

Using expression (58) and the canonical commutation relations for operators Q and P, we obtain the generalized Lindblad equation periodic kicks (57) for Q(t) and P(t) in the form

(60) $$\left(D_{C,0+}^{\alpha }Q\right)\left(t\right)=\frac{1}{m}P\left(t\right)-\lambda Q\left(t\right)\overset{\infty }{\underset{k=1}{\sum }}\delta \left(\frac{t}{T}-k\right),$$

(61) $$\left(D_{C,0+}^{\alpha }P\right)\left(t\right)=-\lambda P\left(t\right)\overset{\infty }{\underset{k=1}{\sum }}\delta \left(\frac{t}{T}-k\right),$$

where

(62) $$\lambda =\lambda_{D}\;Im\left(a_{1}b_{1}^{*}+a_{2}b_{2}^{*}\right).$$

Let us derive exact solutions of these equations and then the quantum dynamical maps with memory. To obtain a solution of the suggested fractional differential equation, we will use the the Riemann–Liouville fractional integral and the second fundamental theorem of fractional calculus.

The left-sided Riemann-Liouville fractional integral is defined by the equation

(63) $$\left(I_{RL;a+}^{\alpha }f\right)\left(t\right)=\frac{1}{\Gamma \left(\alpha \right)}\int_{\alpha }^{t}{\left(t-\tau \right)}^{\alpha -1}f(\tau )d\tau ,$$

where Γ(α) is the gamma function, and the function f(t) satisfies the condition $f\left(t\right)\in L_{1}\left(a,b\right)$. The relationship between the Caputo fractional derivatives and the Riemann–Liouville fractional integrals is described by properties, which are the fundamental theorems of fractional calculus (for example, see Lemmas 2.21 and 2.22 of (pp. ~95–96, [16]). The second fundamental theorem of fractional calculus for these operators is described by Lemma 2.22, which states (p. ~96, [16]) the following: Let α>0, N=[α]+1 for non-integer α and N=α for integer α. If $f\left(t\right)\in AC^{N}\left[a,b\right]$ or $f\left(t\right)\in C^{N}\left[a,b\right]$, then we have the equality

(64) $$\left(I_{RL;a+}^{\alpha }D_{C;a+}^{\alpha }f\right)\left(t\right)=f\left(t\right)-\overset{N-1}{\underset{k=0}{\sum }}\frac{f^{\left(k\right)}\left(a+\right)}{k!}\;{\left(t-a\right)}^{k}.$$

In particular, for 0<α≤1, (N=1), the equation

(65) $$\left(I_{RL;a+}^{\alpha }D_{C;a+}^{\alpha }f\right)\left(t\right)=f\left(t\right)-f\left(a\right)$$

is valid if f(t)∈AC[a,b] or f(t)∈C[a,b].

The application of the Riemann–Liouville fractional integral to Equations (60) and (61) with 0<α≤1, and the use of the second fundamental of fractional calculus gives the equations

(66) $$Q\left(t\right)-Q\left(0\right)=\frac{1}{m}\left(I_{RL,0+}^{\alpha }P\right)\left(t\right)-\lambda \left(I_{RL,0+}^{\alpha }Q\left(\tau \right)\overset{\infty }{\underset{k=1}{\sum }}\delta \left(\frac{\tau }{T}-k\right)\right),$$

(67) $$P\left(t\right)-P\left(0\right)=-\lambda \left(I_{RL,0+}^{\alpha }P\left(\tau \right)\overset{\infty }{\underset{k=1}{\sum }}\delta \left(\frac{\tau }{T}-k\right)\right).$$

Using the definition of the Riemann-Liouville integral (63), Equations (66) and (67) can be writtten as

(68) $$Q\left(t\right)-Q\left(0\right)=\frac{1}{m}\left(I_{RL,0+}^{\alpha }P\right)\left(t\right)-\frac{\lambda }{\Gamma \left(\alpha \right)}\int_{0}^{t}Q\left(\tau \right){\left(t-\tau \right)}^{\alpha -1}\overset{\infty }{\underset{k=1}{\sum }}\delta \left(\frac{\tau }{T}-k\right)d\tau ,$$

(69) $$P\left(t\right)-P\left(0\right)=-\frac{\lambda }{\Gamma \left(\alpha \right)}\int_{0}^{t}P\left(\tau \right){\left(t-\tau \right)}^{\alpha -1}\overset{\infty }{\underset{k=1}{\sum }}\delta \left(\frac{\tau }{T}-k\right)d\tau .$$

For nT<t<(n+1)T, Equations (68) and (69) take the form

(70) $$Q\left(t\right)=Q\left(0\right)+\frac{1}{m}\left(I_{RL,0+}^{\alpha }P\right)\left(t\right)-\frac{\lambda }{\Gamma \left(\alpha \right)}\overset{n}{\underset{k=1}{\sum }}\int_{0}^{t}Q\left(\tau \right){\left(t-\tau \right)}^{\alpha -1}\delta \left(\frac{\tau }{T}-k\right)d\tau ,$$

(71) $$P\left(t\right)=P\left(0\right)-\frac{\lambda }{\Gamma \left(\alpha \right)}\overset{n}{\underset{k=1}{\sum }}\int_{0}^{t}P\left(\tau \right){\left(t-\tau \right)}^{\alpha -1}\delta \left(\frac{\tau }{T}-k\right)d\tau .$$

The Dirac-delta function has the property

(72) $$\int_{0}^{t}f\left(\tau \right)\delta \left(\frac{\tau }{T}-k\right)d\tau =T\;f\left(kT\right)\theta \left(t-kT\right),$$

which holds if 0<kT<t and f(t) is continuous function in τ=kT, where θ(t−kT) is the Heaviside step function, which is equal to zero when k>t/T (i.e., t<kT).

Using this property, Equations (70) and (71) for t∈(nT,(n+1)T), can be written in the form

(73) $$Q\left(t\right)=Q\left(0\right)+\frac{1}{m}\left(I_{RL,0+}^{\alpha }P\right)\left(t\right)-\frac{\lambda T}{\Gamma (\alpha )}\overset{n}{\underset{k=1}{\sum }}Q\left(kT\right){\left(t-kT\right)}^{\alpha -1}\theta (t-kT),$$

(74) $$P\left(t\right)=P\left(0\right)-\frac{\lambda T}{\Gamma \left(\alpha \right)}\overset{n}{\underset{k=1}{\sum }}P\left(kT\right){\left(t-kT\right)}^{\alpha -1}\theta \left(t-kT\right).$$

To obtain expression for $\left(I_{RL,0+}^{\alpha }P\right)\left(t\right)$, we should use Property 2.1 in (p. ~51, [16]) in the form

(75) $$\left(I_{RL,a+}^{\alpha }{\left(\tau -a\right)}^{\beta }\right)\left(t\right)=\frac{\Gamma \left(\beta +1\right)}{\Gamma \left(\alpha +\beta +1\right)}{\left(t-a\right)}^{\alpha +\beta }$$

for t>a, α>0, β>0. In particular,

(76) $$\left(I_{RL,a+}^{\alpha }1\right)\left(t\right)=\frac{1}{\Gamma \left(\alpha +1\right)}{\left(t-a\right)}^{\alpha }.$$

Therefore, for nT<t<(n+1)T, the action of the Riemann–Liouville fractional integral on Equation (74) gives

(77) $$\left(I_{RL,0+}^{\alpha }P\right)\left(t\right)=P\left(0\right)\left(I_{RL,0+}^{\alpha }1\right)\left(t\right)-\;\frac{\lambda_{T}}{\Gamma \left(\alpha \right)}\overset{n}{\underset{k=1}{\sum }}P(kT)(I_{RL,0+}^{\alpha }{\left(\tau -kT\right)}^{\alpha -1}\theta \left(\tau -kT)(t)=\;P\left(0\right)(I_{RL,0+}^{\alpha }1)(t)-\frac{\lambda T}{\Gamma (\alpha )}\overset{n}{\underset{k=1}{\sum }}P\left(kT\right)(I_{RL,kT+}^{\alpha }{\left(\tau -kT\right)}^{\alpha -1}\right)(t)=\;P\left(0\right)\frac{1}{\Gamma \left(\alpha +1\right)}\tau^{\alpha }-\frac{\lambda T}{\Gamma \left(\alpha \right)}\overset{n}{\underset{k=1}{\sum }}P(kT)\frac{\Gamma (\alpha )}{\Gamma \left(2\alpha \right)}{\left(t-kT\right)}^{2\alpha -1}\theta (t-kT)=\;P\left(0\right)\frac{1}{\Gamma \left(\alpha +1\right)}\tau^{\alpha }-\frac{\lambda T}{\Gamma \left(2\alpha \right)}\overset{n}{\underset{k=1}{\sum }}P(kT){\left(t-kT\right)}^{2\alpha -1}\theta (t-kT),$$

where we use $I_{RL,0+}^{\alpha }\;\theta \left(t-kT\right)=I_{RL,kT+}^{\alpha }$.

Substitution of expression (77) into Equation (73) gives the equations for coordinate operator Q(t).

As a result, we proved the following Proposition.

**Proposition** **2.** *The solutions of generalized Lindblad equations for coordinate (60) and momentum (61) at* t∈(nT,(n+1)T) has the form

(78) $$Q\left(t\right)=Q\left(0\right)+P\left(0\right)\frac{1}{m\Gamma \left(\alpha +1\right)}t^{\alpha }-\;\frac{\lambda \;T}{m\Gamma \left(2\alpha \right)}\overset{n}{\underset{k=1}{\sum }}P\left(kT\right){\left(t-kT\right)}^{2\alpha -1}\theta \left(t-kT\right)-\;\frac{\lambda \;T}{\Gamma \left(\alpha \right)}\overset{n}{\underset{k=1}{\sum }}Q\left(kT\right){\left(t-kT\right)}^{\alpha -1}\theta \left(t-kT\right),$$

(79) $$P\left(t\right)=P\left(0\right)-\frac{\lambda \;T}{\Gamma \left(\alpha \right)}\overset{n}{\underset{k=1}{\sum }}P\left(kT\right){\left(t-kT\right)}^{\alpha -1}\theta \left(t-kT\right),$$

*where* θ(z) *is the Heaviside step function.*

Equations (78) and (79) are exact solutions of the generalized Lindblad Equations (60) and (61) for 0<α≤1 and t∈(0,(n+1)T). These solutions describe the non-Markovian dynamics of an open quantum system with power-law memory.

Let us derive expressions for the non-Markovian quantum dynamics in the form of quantum discrete-time maps with memory. For the left side of the (n+1)th kicks (t=(n+1)T−ε), where

(80) $$Q_{n+1}=\underset{\varepsilon \to 0+}{\lim}Q\left(T\left(n+1\right)-\varepsilon \right),$$

(81) $$P_{n+1}=\underset{\varepsilon \to 0+}{\lim}P\left(T\left(n+1\right)-\varepsilon \right),$$

solutions (78) and (79) are given by the equations

(82) $$Q_{n+1}=Q_{0}+P_{0}\frac{T^{\alpha }}{m\Gamma \left(\alpha +1\right)}{\left(n+1\right)}^{\alpha }-\;\frac{\lambda \;T^{2\alpha }}{m\Gamma \left(2\alpha \right)}\overset{n}{\underset{k=1}{\sum }}P_{k}\;{\left(n+1-k\right)}^{2\alpha -1}-\frac{\lambda \;T^{\alpha }}{\Gamma \left(\alpha \right)}\overset{n}{\underset{k=1}{\sum }}Q_{k}\;{\left(n+1-k\right)}^{\alpha -1},$$

(83) $$P_{n+1}=P_{0}-\frac{\lambda \;T^{\alpha }}{\Gamma \left(\alpha \right)}\overset{n}{\underset{k=1}{\sum }}P_{k}{\left(n+1-k\right)}^{\alpha -1}.$$

For the left side of the n-th kicks (t=nT−ε), we have

(84) $$Q_{n}=Q_{0}+P_{0}\frac{T^{\alpha }}{\Gamma \left(\alpha +1\right)}n^{\alpha }-\;\frac{\lambda \;T^{2\alpha }}{m\Gamma \left(2\alpha \right)}\overset{n-1}{\underset{k=1}{\sum }}P_{k}{\left(n-k\right)}^{2\alpha -1}-\frac{\lambda \;T^{\alpha }}{\Gamma \left(\alpha \right)}\overset{n-1}{\underset{k=1}{\sum }}Q_{k}{\left(n-k\right)}^{\alpha -1},$$

(85) $$P_{n}=P_{0}-\frac{\lambda T^{\alpha }}{\Gamma \left(\alpha \right)}\overset{n-1}{\underset{k=1}{\sum }}P_{k}\;{\left(n-k\right)}^{\alpha -1}.$$

Subtracting from the expressions for n+1 the expressions for n, we obtain the discrete-time quantum maps with memory.

As a result, we proved the Proposition that describes the quantum discrete map with memory.

**Proposition** **3.** *The solutions of generalized Lindblad equations for coordinate (60) and momentum (61) can be represented as the quantum discrete-time map with memory in the form*

(86) $$Q_{n+1}=Q_{n}+P_{0}\frac{T^{\alpha }}{\Gamma \left(\alpha +1\right)}V_{\alpha +1}\left(n\right)-\frac{\lambda T^{2\alpha }}{m\Gamma \left(2\alpha \right)}P_{n}-\frac{\lambda T^{2\alpha }}{m\Gamma \left(2\alpha \right)}\overset{n-1}{\underset{k=1}{\sum }}P_{k}V_{2\alpha }\left(n-k\right)-\;\frac{\lambda T^{\alpha }}{\Gamma \left(\alpha \right)}Q_{n}-\frac{\lambda T^{\alpha }}{\Gamma \left(\alpha \right)}\overset{n-1}{\underset{k=1}{\sum }}Q_{k}V_{\alpha }\left(n-k\right),$$

(87) $$P_{n+1}=P_{n}-\frac{\lambda T^{\alpha }}{\Gamma \left(\alpha \right)}P_{n}-\frac{\lambda T^{\alpha }}{\Gamma \left(\alpha \right)}\overset{n-1}{\underset{k=1}{\sum }}P_{k}V_{\alpha }\left(n-k\right),$$

*where we use the function*

(88) $$V_{\alpha }\left(z\right)={\left(z+1\right)}^{\alpha -1}-z^{\alpha -1},$$

*where* z>0 *.*

It should be emphasized that the proposed discrete maps with memory are obtained from the generalized Lindblad equations for Q and P without using any approximations.

As a result, we can see that the quantum dynamical maps (86), (87) with α∉N are maps with fading memory. The memory means that evolution of the quantum observable

(89) $$A\left(t_{n+1}\right)=\Phi_{n+1}\left(\alpha \right)A\left(0\right)$$

depends on all past values of $A\left(t_{k}\right)$ for k≤n, ($t_{k}<t_{n+1}$).

## 5. Nonlinear Quantum Map with Memory from Generalized Lindblad Equation

Let us consider an example of nonlinear quantum map that can be derived from the generalized Lindblad equation and periodic kicks for coordinate and momentum operators.

Let us consider the non-Markovian master equation for an open quantum system with power-law memory and periodic kicks in the form (57), where the Lindblad operators $V_{k}=V_{k}\left(Q,P\right)$ and the Hamiltonian H=H(Q,P) have the form

(90) $$H=\frac{1}{2m}P^{2},\;V_{k}=a_{k}F\left(P\right)+b_{k}G\left(Q\right),$$

where $a_{k}$, and $b_{k}$, k=1,2, are complex numbers, F(P) and G(Q) are entire functions or polynomials of the coordinate and momenta operators, and 0<α≤1.

The generalized Lindblad equations (90) with power-law memory and periodic kicks for the coordinate and momentum have the forms

(91) $$\left(D_{C,0+}^{\alpha }Q\right)\left(t\right)=\frac{1}{m}P\left(t\right)-\lambda_{D}\;D_{V}\left[Q\left(t\right)\right]\overset{\infty }{\underset{k=1}{\sum }}\delta \left(\frac{t}{T}-k\right),.\;$$

(92) $$\left(D_{C,0+}^{\alpha }P\right)\left(t\right)=\lambda_{D}\;D_{V}\left[P\left(t\right)\right]\overset{\infty }{\underset{k=1}{\sum }}\delta \left(\frac{t}{T}-k\right).\;$$

Using that

(93) $$L_{V}\Phi_{t}(\alpha )\;=\;\Phi_{t}(\alpha )L_{V},$$

we obtain for Equations (91) and (92) in the form

(94) $$D_{V}\left[Q\left(t\right)\right]=D_{V}\left[\Phi_{t}\left(\alpha \right)\;Q\right]=\Phi_{t}\left(\alpha \right)\;D_{V}\left[Q\right]=D_{V}\left[Q\right]\left(t\right),$$

(95) $$D_{V}\left[P\left(t\right)\right]=D_{V}\left[\Phi_{t}\left(\alpha \right)\;P\right]=\Phi_{t}\left(\alpha \right)\;D_{V}\left[P\right]=D_{V}\left[P\right]\left(t\right).$$

Because of this, we can substitute expressions (90) into Equation (58) to obtain an explicit form of dissipators for the coordinate and momentum operators. Using the expression (90), the definition of $D_{V}$ and the canonical commutation relations for operators Q and P, we obtain the expressions

(96) $$D_{V}\left[Q\right]=\frac{1}{2\hbar }\left(\lambda_{a}\;[F\left(P\right),[Q,F\left(P\right)\right]]+Re\left(\lambda_{ab}\right)\;[G\left(Q\right),[Q,F\left(P\right)]]+\;i\;Im\left(\lambda_{ab}\right)\;{\left\{G\left(Q\right),\left[Q,F\left(P\right)\right]\right\}}_{+}),$$

(97) $$D_{V}\left[P\right]=\frac{1}{2\hbar }\left(\lambda_{b}\;[G\left(Q\right),[P,G\left(Q\right)\right]]+Re\left(\lambda_{ab}\right)\;[F\left(P\right),[P,G\left(Q\right)]]-\;i\;Im\left(\lambda_{ab}\right)\;{\left\{F\left(P\right),\left[P,G\left(Q\right)\right]\right\}}_{+}),$$

where

(98) $${\left\{A,B\right)}_{+}=AB+BA,$$

(99) $$\lambda_{a}=\overset{2}{\underset{k=1}{\sum }}a_{k}a_{k}^{*},\lambda_{b}=\overset{2}{\underset{k=1}{\sum }}b_{k}b_{k}^{*},\lambda_{ab}=\overset{2}{\underset{k=1}{\sum }}a_{k}b_{k}^{*}.$$

For example, if we use the functions

(100) F(P)=P, G(Q)=Q,

then Equations (96) and (97) take form

(101) $$D_{V}\left[Q\right]=-Im\left(\lambda_{ab}\right)\;Q,$$

(102) $$D_{V}\left[P\right]=-Im\left(\lambda_{ab}\right)\;P.$$

In this case, Equations (91) and (92) give Equations (60) and (61).

If we use the functions

(103) $$F\left(P\right)=P,\;G\left(Q\right)=Q^{2},$$

then Equations (96) and (97) take the form

(104) $$D_{V}\left[Q\right]=-Im\left(\lambda_{ab}\right)\;Q^{2},$$

(105) $$D_{V}\left[P\right]=-\hbar \;Re\left(\lambda_{ab}\right)+Im\left(\lambda_{ab}\right)\;\left(Q\;P+P\;Q\right).$$

The application of the Riemann–Liouville fractional integral to Equations (91) and (92) with 0<α≤1, and the second fundamental of fractional calculus, gives the equations

(106) $$Q\left(t\right)-Q\left(0\right)=\frac{1}{m}\left(I_{RL,0+}^{\alpha }P\right)\left(t\right)-\lambda_{D}\left(I_{RL,0+}^{\alpha }+D_{V}\left[Q\right]\left(\tau \right)\overset{\infty }{\underset{k=1}{\sum }}\delta \left(\frac{\tau }{T}-k\right)\right),$$

(107) $$P\left(t\right)-P\left(0\right)=-\lambda \left(I_{RL,0+}^{\alpha }D_{V}\left[P\right]\left(\tau \right)\overset{\infty }{\underset{k=1}{\sum }}\delta (\frac{\tau }{T}-k)\right)$$

Using the definition of the Riemann–Liouville integral (63), we have

(108) $$Q\left(t\right)-Q\left(0\right)=\frac{1}{m}\left(I_{RL,0+}^{\alpha }P\right)\left(t\right)-\;\frac{\lambda_{D}}{\Gamma \left(\alpha \right)}\int_{0}^{t}D_{V}\left[Q\right]\left(\tau \right){\left(t-\tau \right)}^{\alpha -1}\overset{\infty }{\underset{k=1}{\sum }}\delta (\frac{\tau }{T}-k)d\tau ,$$

(109) $$P\left(t\right)-P\left(0\right)=-\frac{\lambda_{D}}{\Gamma \left(\alpha \right)}\int_{0}^{t}D_{V}\left[P\right]\left(\tau \right){\left(t-\tau \right)}^{\alpha -1}\overset{\infty }{\underset{k=1}{\sum }}\delta (\frac{\tau }{T}-k)d\tau .$$

Carrying out the transformations performed in the previous section, we can obtain the solution of the nonlinear equations for the coordinate and momentum. As a result, we have the solution of the considered equations for t∈(nT,(n+1)T) in the form

(110) $$Q\left(t\right)=Q\left(0\right)+P\left(0\right)\frac{1}{\Gamma \left(\alpha +1\right)}t^{\alpha }-\frac{\lambda_{D}T}{m\Gamma \left(2\alpha \right)}\overset{n}{\underset{k=1}{\sum }}D_{V}\left[P\right]\left(kT\right)\;{\left(t-kT\right)}^{2\alpha -1}-\;\frac{\lambda_{D}T}{\Gamma \left(\alpha \right)}\overset{n}{\underset{k=1}{\sum }}D_{V}\left[P\right]\left(kT\right)\;{\left(t-kT\right)}^{\alpha -1},$$

(111) $$P\left(t\right)=P\left(0\right)--\frac{\lambda_{D}T}{\Gamma \left(\alpha \right)}\overset{n}{\underset{k=1}{\sum }}D_{V}\left[Q\right]\left(kT\right)\;{\left(t-kT\right)}^{\alpha -1},$$

Let us use the coordinate and momentum for the left side of the k-th kicks (t=kT−ε), where

(112) $$Q_{k}=\underset{\varepsilon \to 0+}{\lim}Q\left(kT-\varepsilon \right),\;P_{k}=\underset{\varepsilon \to 0+}{\lim}P\left(kT-\varepsilon \right)$$

with k∈N.

Using the transformations performed in the previous section, we attain the quantum discrete-time map with memory.

As a result, we obtain the Proposition that describes the nonlinear quantum discrete-time map with memory.

**Proposition** **4.** *The solutions of generalized Lindblad equations for coordinate (91), (96) and momentum (92), (97) can be represented as the quantum discrete-time map with memory in the form*

(113) $$Q_{n+1}=Q_{n}+P_{0}\frac{T^{\alpha }}{m\Gamma \left(\alpha +1\right)}V_{\alpha +1}\left(n\right)-\;\frac{\lambda_{D}T^{2\alpha }}{m\Gamma \left(2\alpha \right)}D_{n}\left[P\right]-\frac{\lambda_{D}T^{2\alpha }}{m\Gamma \left(2\alpha \right)}\overset{n-1}{\underset{k=1}{\sum }}D_{k}\left[P\right]V_{2\alpha }\left(n-k\right)-\;\frac{\lambda_{D}T^{\alpha }}{\Gamma \left(\alpha \right)}D_{n}\left[Q\right]-\frac{\lambda_{D}T^{\alpha }}{\Gamma \left(\alpha \right)}\overset{n}{\underset{k=1}{\sum }}D_{k}\left[Q\right]\;V_{\alpha }\left(n-k\right),$$

(114) $$P_{n+1}=P_{n}-\frac{\lambda_{D}T^{\alpha }}{\Gamma \left(\alpha \right)}D_{n}\left[P\right]-\frac{\lambda_{D}T^{\alpha }}{\Gamma \left(\alpha \right)}\overset{n}{\underset{k=1}{\sum }}D_{k}\left[P\right]\;V_{\alpha }\left(n-k\right),$$

where

(115) $$D_{k}\left[Q\right]=\underset{\varepsilon \to 0+}{lim}D_{V}\left[Q\right]\left(kT-\varepsilon \right),$$

(116) $$D_{k}\left[P\right]=\underset{\varepsilon \to 0+}{lim}D_{V}\left[P\right]\left(kT-\varepsilon \right)$$

*with* k=1,…,n *and* T>0*.*

We should emphasize that, in general, we have the inequalities

(117) $$D_{V}\left[Q\right]\left(t\right)\neq D_{V}\left[Q\left(t\right)\right],\;D_{V}\left[P\right]\left(t\right)\neq D_{V}\left[P\left(t\right)\right],$$

where

(118) $$D_{V}\left[A\right]\left(t\right)=\Phi_{t}\left(\alpha \right)\left(D_{V}\left[A\right]\right),\;D_{V}\left[A\left(t\right)\right]=D_{V}\left[\Phi_{t}\left(\alpha \right)A\right],$$

since, for open quantum systems

(119) $$\Phi_{t}\left(\alpha \right)\left(A\;B\right)\neq \Phi_{t}\left(\alpha \right)\left(A\right){\;\Phi }_{t}\left(\alpha \right)\left(B\right)$$

for t>0, in general, i.e., (AB)(t)≠A(t)B(t). In particular, we have

(120) $$\Phi_{t}\left(\alpha \right)\left(A^{2}\right)\neq {\left(\Phi_{t}\left(\alpha \right)A\right)}^{2},\;\mathrm{or}\;\left(A^{2}\right)\left(t\right)\neq {\left(A\left(t\right)\right)}^{2}.$$

An important characteristic of open quantum systems is the following: the evolution of the product of operators does not coincide with the product of the evolved operators. This property is independent of the presence or absence of memory. The property is due to the fact that the Lindblad superoperator $L_{V}$ is not a derivative operator on the space of operators (for example, see [9,12,53]).

As a result, we have the inequalities

(121) $$D_{k}\left[Q\right]\neq \frac{1}{2\hbar }\left(\lambda_{a}\;[F\left(P_{k}\right),[Q_{k},F\left(P_{k}\right)\right]]+\;Re\left(\lambda_{ab}\right)\;[G\left(Q_{k}\right),[Q_{k},F\left(P_{k}\right)]]+i\;Im\left(\lambda_{ab}\right)\;{\left\{G\left(Q_{k}\right),\left[Q_{k},F\left(P_{k}\right)\right]\right\}}_{+},$$

(122) $$D_{k}\left[P_{k}\right]\neq \frac{1}{2\hbar }\left(\lambda_{b}\;[G\left(Q_{k}\right),[P_{k},G\left(Q_{k}\right)\right]]+\;Re\left(\lambda_{ab}\right)\;[F\left(P_{k}\right),[P_{k},G\left(Q_{k}\right)]]-i\;Im\left(\lambda_{ab}\right)\;{\left\{F\left(P_{k}\right),\left[P_{k},G\left(Q_{k}\right)\right]\right\}}_{+}),$$

where k∈N.

For example, if we use the functions F(P)=P and $G\left(Q\right)=Q^{2}$, then Equations (121) and (122) take the form

(123) $$D_{k}\left[Q\right]=-Im\left(\lambda_{ab}\right)\;{\left(Q^{2}\right)}_{k},$$

(124) $$D_{k}\left[P_{k}\right]=-\hbar \;Re\left(\lambda_{ab}\right)+Im\left(\lambda_{ab}\right)\;{\left(QP+PQ\right)}_{k}.$$

where

(125) $${\left(Q^{2}\right)}_{k}=\underset{\varepsilon \to 0+}{\lim}\left(Q^{2}\right)\left(kT-\varepsilon \right)=\underset{\varepsilon \to 0+}{\lim}\Phi_{kT-\varepsilon }\left(\alpha \right)\left(Q^{2}\right),$$

(126) $${\left(QP+PQ\right)}_{k}=\underset{\varepsilon \to 0+}{\lim}\left(QP+PQ\right)\left(kT-\varepsilon \right)=\underset{\varepsilon \to 0+}{\lim}\Phi_{kT-\varepsilon }\left(\alpha \right)\left(QP+PQ\right).$$

Note that we should take into account the inequality

(127) $${\left(Q^{2}\right)}_{k}\neq {\left(Q_{k}\right)}^{2},\;{\left(QP+PQ\right)}_{k}\neq Q_{k}P_{k}+P_{k}Q_{k}$$

To have the expressions ${\left(Q^{2}\right)}_{k}$ and ${\left(QP+PQ\right)}_{k}$, we should consider the generalized Lindblad Equation (57) and the superoperator (5) for $A=Q^{2}$ and A=QP+PQ. In the nonlinear case, we have a system of interconnected equations (chain of equations).

The quantum dynamical maps with α∉N are maps with fading memory, since behavior of the quantum observables on the (n+1)-step $A\left(t_{n+1}\right)=\Phi_{n+1}\left(\alpha \right)A\left(0\right)$ depends on all past k-step values for k≤n.

We should emphasize that the proposed dynamical maps are nonlinear. Therefore, we can assume that it may exhibit chaotic behavior.

## 6. Conclusions

In this paper, we consider non-Markovian dynamics of open quantum systems with the power-law memory and periodic kicks. Non-Markovian generalizations of the Lindblad equations are suggested in the form of fractional differential operator equations with the derivative of non-integers with respect to time. The exact solution of the proposed generalized Lindblad equations for coordinate and momentum operators are derived. We assumed that fractional differential operator equations have found many applications in the construction of non-Markovian theory of open quantum systems and quantum processes with fading memory.

We assume that proposed discrete quantum maps with memory can find different applications in quantum dynamics with memory.

It is safe to hope that the proposed quantum maps with memory can simplify simulations of the behavior of non-Markovian quantum dynamics with power-law fading memory in computer simulations. However, this modeling remains an open question. Hopefully, it will be solved in future research.

## Data Availability

Data sharing not applicable.
